# Feasibility of a Sensor-Based Gait Event Detection Algorithm for Triggering Functional Electrical Stimulation during Robot-Assisted Gait Training

**DOI:** 10.3390/s19214804

**Published:** 2019-11-05

**Authors:** Andreas Schicketmueller, Georg Rose, Marc Hofmann

**Affiliations:** 1 HASOMED GmbH, Paul-Ecke-Str. 1, Magdeburg 39114, Germany; marc.hofmann@hasomed.de; 2 Institute for Medical Engineering and Research Campus STIMULATE, University of Magdeburg Universitaetsplatz 2, Magdeburg 39106, Germany; Georg.Rose@ovgu.de

**Keywords:** FES, IMU, gait event detection, algorithm, hybrid robotic rehabilitation system

## Abstract

Technologies such as robot-assisted gait trainers or functional electrical stimulation can improve the rehabilitation process of people affected with gait disorders due to stroke or other neurological defects. By combining both technologies, the potential disadvantages of each technology could be compensated and simultaneously, therapy effects could be improved. Thus, an algorithm was designed that aims to detect the gait cycle of a robot-assisted gait trainer. Based on movement data recorded with inertial measurement units, gait events can be detected. These events can further be used to trigger functional electrical stimulation. This novel setup offers the possibility of equipping a broad range of potential robot-assisted gait trainers with functional electrical stimulation. The aim of this paper in particular was to test the feasibility of a system using inertial measurement units for gait event detection during robot-assisted gait training. Thus, a 39-year-old healthy male adult executed a total of six training sessions with two robot-assisted gait trainers (Lokomat and Lyra). The measured data from the sensors were analyzed by a custom-made gait event detection algorithm. An overall detection rate of 98.1% ± 5.2% for the Lokomat and 94.1% ± 6.8% for the Lyra was achieved. The mean type-1 error was 0.3% ± 1.2% for the Lokomat and 1.9% ± 4.3% for the Lyra. As a result, the setup provides promising results for further research and a technique that can enhance robot-assisted gait trainers by adding functional electrical stimulation to the rehabilitation process.

## 1. Introduction

The human gait is a complex interaction between the human sensory and motor systems and counts as one of the most important activities of daily living. Numerous pathologies such as stroke, spinal cord injury, and multiple sclerosis can disrupt the finely adjusted system and can cause gait disorders or the incapability of walking. In order to counteract gait disorders, various rehabilitation techniques are used in clinical routine. Besides conventional physiotherapy, electromechanical gait training/robot-assisted gait training is used. The used gait trainers provide a harness and a body weight support system that supports the paretic lower limbs. Additionally, it compensates for the lack of balance reactions and it supports the patient in case of obesity. The gait pattern is externally induced by a robot-specific strategy. Robot-assisted gait trainers such as the Gait Trainer GT II (Reha Stim, Berlin, Germany), G-EO (Reha Technology AG, Olten, Switzerland), Lyra (Thera Trainer, Hochdorf, Germany), and the Lokomat (Hocoma, Volketswil, Switzerland) are used in clinical routine. These robots can be classified depending on their movement principle into two main groups. Exoskeletons move joints such as the knee and ankle joint during the gait cycle, whereas controllers of endpoint trajectories move only the feet. Typical examples of these robots are the Lokomat as an exoskeleton and the Gait Trainer (or Gang Trainer) as end-effector based system [1]. A current systematic review of electromechanical-assisted training for walking after stroke concludes that people who receive electromechanical-assisted gait training in combination with physiotherapy after stroke are more likely to achieve independent walking than people who receive gait training without these devices [2]. One drawback of robotic orthosis is that it leads to changes in naturally occurring muscle activation patterns [3]. Additionally, these devices only provide passive movement to the lower limbs, and the body weight support system partially inhibits muscle activity [4]. To counteract the deficit of improper muscle activation, functional electrical stimulation (FES) can be used to activate the muscle by applying electrical stimulation to the muscle or the according nerve. FES is generally referred to as an artificial electrical stimulation of a muscle that has a diminished nervous control, with the aim of providing muscular contractions and producing functionally useful movements [5]. Current evidence shows that FES has positive orthotic effects on people with multiple sclerosis [6]. Nevertheless, therapeutic effects were not significant and not all patients are suitable for FES, which concludes that innovation and advanced clinical approaches may increase FES usability and efficacy [6]. For stroke patients, it has been shown that FES gait training is superior to conventional electrical stimulation (electromyostimulation). FES improved mobility and balance as well as gait performance and reduced spasticity [7]. For spinal cord injury patients, similarly significant quality of life improvements such as cardiovascular conditioning can be achieved and maintained [8]. For the further improvement of rehabilitation, these techniques (robot-assisted gait training and FES) can be used together in order to partially compensate for each other’s disadvantages. The combination of these technologies is conventionally termed hybrid robotic rehabilitation systems, which are defined as systems that aim to achieve motor recovery or compensate for motor function by combining electrically-stimulated muscle action and torque provision to the joints [9] they are also more effective than robot-assisted gait training alone [10]. Approaches to combine systems such as the Lokomat with FES intramuscular electrodes are promising; nevertheless, they either focus on intramuscular electrodes and information provided by the Lokomat itself [11,12] or they focused on foot drop with a limited amount of stimulated muscles [13]. In order to overcome the dependencies on the information provided by the robot, inertial measurement units (IMUs) can be used to detect the gait cycle with the further aim of triggering functional electrical stimulation. This approach results in a bigger variety of potential gait trainers and can improve the amount of stimulated muscles due to the exact knowledge of the gait events. Gait analysis systems such as the RehaGait (HASOMED GmbH, Magdeburg, Germany) or the PhysiGait (Gait UP S.A., Lausanne, Switzerland) using IMUs are already available on the market and have shown to be valid and reliable [14,15,16,17]. Yet, these systems do not aim to trigger electrical stimulation. They were developed to analyze the human gait rather than the robot-induced gait pattern with its system-dependent peculiarities in the movement. Here, we present a novel technique that combines gait detection from IMU signals and robot-assisted gait training with the aim of supporting the rehabilitation with optimally triggered FES.

## 2. Materials and Methods 

The overall concept of the system aims to be used as an add-on for a robot-assisted gait trainer such as Lokomat or Lyra, thus creating a hybrid robotic rehabilitation system. The system deals with specific scientific and technological challenges, including:

- Real-time gait detection from an easy-to-use system setup
- Arbitrary sensor alignment
- Functional electrical stimulation during robot-assisted gait training
- Despite introducing new technologies, keeping the additional time expenditure for the therapist on a low level

**Figure 1:**
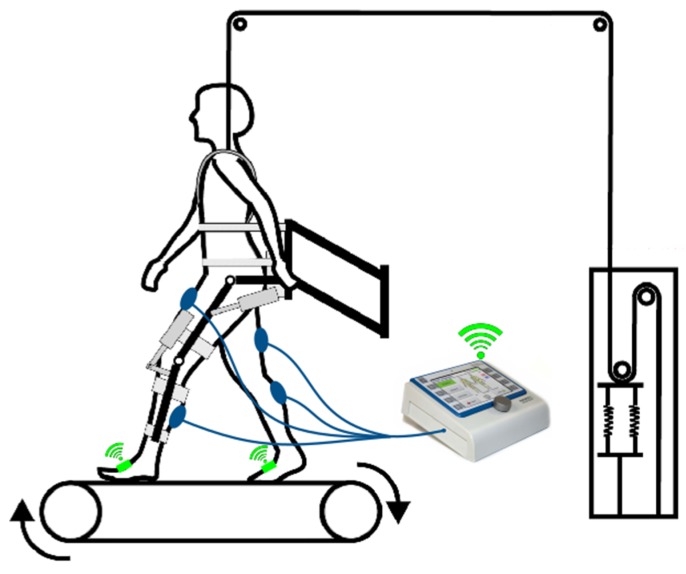
Concept: Lokomat with inertial measurement units (IMUs) and functional electrical stimulation (FES), adapted from [19].

**Figure 2:**
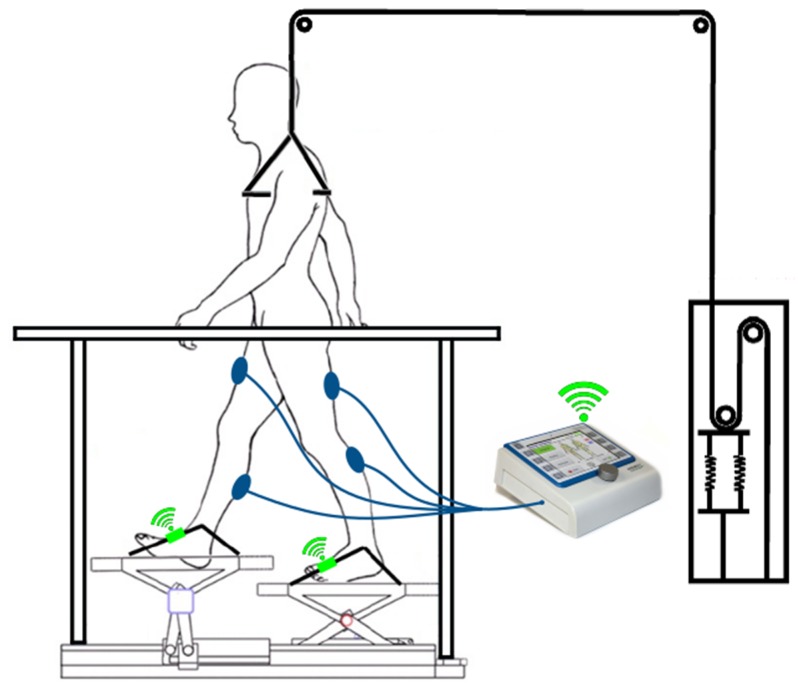
Concept: Lyra with IMUs and FES, adapted from [21]

The overall concepts are shown in Figure 1 and Figure 2. The individual components including the developed algorithm for the gait detection are described in detail in the following sections.

### 2.1. Robot-Assisted Gait Trainer

**Lokomat:** The Lokomat is a commercially available robot-assisted gait trainer that is composed of robotic gait orthosis, a body weight support system, and a treadmill. Programmable actuators in the orthosis enable a cyclic gait pattern. The system is a state-of-the-art gait rehabilitation system [2] and provides effective gait training [18].

**Lyra:** The Lyra is an end-effector based robot-assisted gait trainer. It provides a physiological gait pattern and a short setup time due to the ground-level entrance of the system, which allows easy accessibility for wheelchairs. For stroke patients, gait trainers with the end-effector principle yielded a superior effect on gait function, balance, and activities of daily living compared to conventional gait training [20].

### 2.2. Electrical Stimulator

The RehaStim (RehaStim, HASOMED GmbH, Magdeburg, Germany) is a portable electrical stimulation device that generates impulses on up to eight channels simultaneously. Surface electrodes are used to activate paralyzed muscles. This stimulator is certified according to the EU guideline EN60601-2-10 for medical technical devices and systems. The device can be used alone or in combination with external processing units and has been used as stimulator in recent research [22].

RehaStim is capable of providing currents up to 130 mA with a step size of 5 mA; the frequency can be set from 10 Hz to 50 Hz with a step size of 5 Hz. The according pulse width can be adjusted from 20 μs up to 500 μs with a step size of 10 μs. The stimulator provides biphasic rectangular impulses in order to provide charge neutrality within the stimulated area. Thus, a large variety of different settings is provided, which gives clinicians the capability of adjusting the parameters according to the needs of the patient.

### 2.3. Inertial Measurement Unit

Data acquisition was realized with two custom-built inertial measurement units (MotionSensor, HASOMED GmbH, Magdeburg, Germany). Each IMU consisted of a three-axis accelerometer (±16 g) and a three-axis gyroscope (± 2000°/s) [15]. The sampling frequency was set to 500 Hz, and the synchronization of the IMUs was realized by utilizing a Bluetooth clock. 

### 2.4. Automatic Real-Time Gait Detection Algorithm

**Figure 3:**
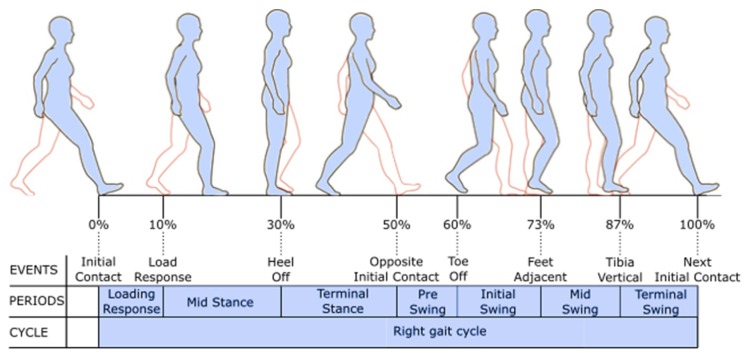
Gait cycle [23].

The human gait cycle consists of two phases: the stance phase and the swing phase. These phases are determined by certain gait events (Figure 3). Gait trainers aim at providing a physiological gait pattern; thus, the concept of human gait is used for the gait event detection.

**Figure 4:**
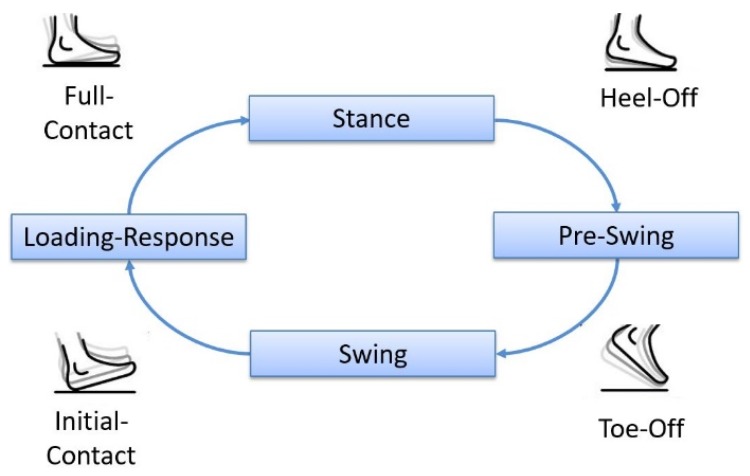
Finite-state diagram of gait events, adapted from [24].

As not all gait events are reliably detectable with IMUs, the presented algorithm focuses on the most important gait events for defining the gait cycle. These events are the Initial Contact, Full Contact during Midstance, Heel Off, and the Toe Off. With two IMUs, these gait events are detectable for each lower limb, resulting in an amount of eight detectable events during one full gait cycle. As gait trainers provide a cyclic movement resulting in a cyclic gait, unwanted switching between the gait phases (e.g., no proper Heel Off due to gait disorders during walking) can be excluded. Additionally, gait trainers limit the movement to the sagittal plane. Taking these boundary conditions into account, a finite-state diagram as shown in Figure 4 can be inferred.

#### 2.4.1. Arbitrary Sensor Alignment Algorithm

**Figure 5:**
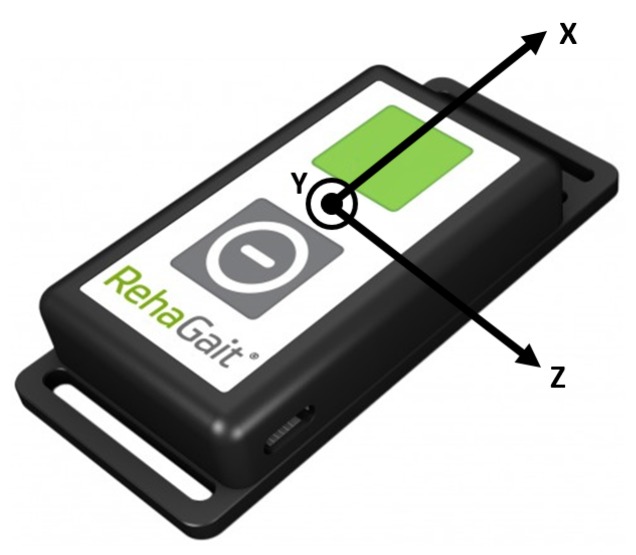
Local coordinate system of an IMU.

In order to detect the gait events, one IMU per lower limb is used. Inertial measurement units measure their data in their local coordinate system (Figure 5).

For further processing of the gathered data, the local coordinate system of the IMU has to be aligned with the local coordinate system of the foot. This can be done by two approaches:

- Tightly attaching the sensor to the foot and overlapping the two coordinate systems manually.
- Calculating the current orientation of the sensor for each data point using an arbitrary sensor alignment algorithm.

The latter option is more robust against changes of IMU position, which can happen during robot-assisted gait training. Additionally, it allows an arbitrary sensor positioning, which makes it easier for the therapist to use, as they do not have focus on the correct positioning of the IMU. Furthermore, the orthosis of the robot-assisted gait trainer is rigidly attached to the patient’s leg; as a result, a pre-defined sensor position—as mentioned in the first option—could interfere with the attachments of the orthosis, making it impossible to realise a feasible setup.

**Figure 6:**
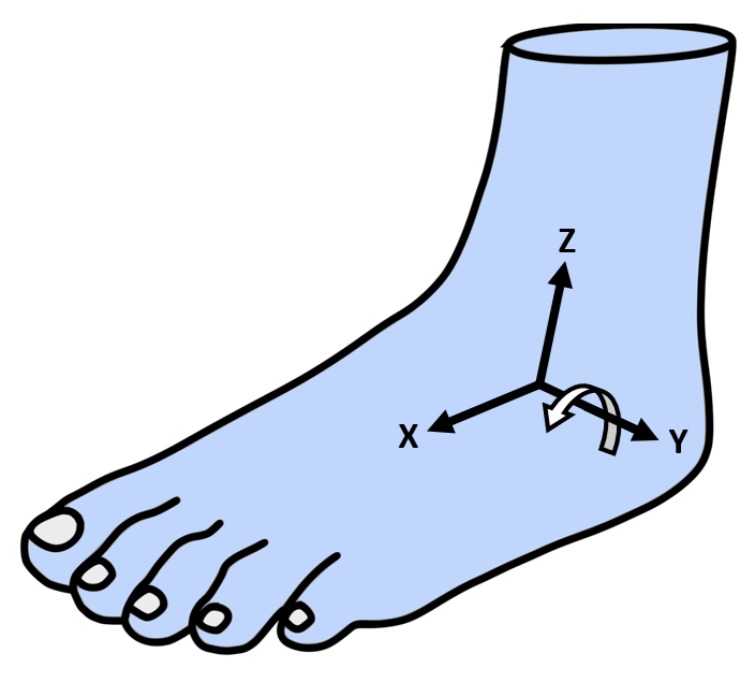
Local axis of the left foot.

For realizing an arbitrary sensor positioning, the local coordinate system of the sensor (Figure 5) must be aligned with the coordinate system of the foot (Figure 6) by a mathematical operation (similar approach to that mentioned in [24]. Thus, a rotation matrix that represents the contortion between the coordinate systems has to be established. The rotation matrix as defined in Equation (1) is determined in four steps:

(1) $$RotationMatrix=\;\left(\begin{array}{ccc} x_{axis\;x} & y_{axis\;x} & z_{axis\;x}\\ x_{axis\;y} & y_{axis\;y} & z_{axis\;y}\\ x_{axis\;z} & y_{axis\;z} & z_{axis\;z} \end{array}\right)$$

(1) Full Contact detection and ${\vec{z}}_{axis}$ determination

During the cyclic movement of the gait trainer, Full Contact can be detected when the angular velocity ${\vec{\omega }}_{FullContact\;}$ remains in a small range fulfilling all three following conditions:

(2) $$\begin{array}{r} |\omega_{\;x}\left(i\right)|<\omega_{FullContact\;tresh\;}\\ |\omega_{\;y}\left(i\right)|<\omega_{FullContact\;tresh}\\ |\omega_{\;z}\left(i\right)|<\omega_{FullContact\;tresh}\\ \forall \;i\in \left(n\ldots .\;k\right) \end{array}$$

where *n* denotes the current sample, *k* denotes a reasonable amount of passed samples, and $\omega_{FullContact\;tresh}$ represents the threshold value for the Full Contact detection.

When the Full Contact condition is satisfied, the ${\vec{z}}_{axis}$ of the rotation matrix is in accordance with the acceleration vector ${\vec{a}}_{FullContact}$. Thus, the ${\vec{z}}_{axis}$ represents the current tilt of the sensor in its local coordinate system.

(3) $${\vec{z}}_{axis}=\;\left(\begin{array}{c} a_{FullContact\;x}\\ a_{FullContact\;y}\\ a_{FullContact\;z} \end{array}\right)$$

(2) Defining the main rotation axis and ${\vec{y}}_{axis}$ determination

After Midstance, the heel starts to rise (Figure 3), causing a rotation in the local ${\vec{y}}_{axis}\;$ of the foot (Figure 6).

After Midstance has passed, the main rotation axis of the IMU can be determined by summing up the angular velocity of each axis starting with the first sample *n* after Full Contact, and ending after a certain amount of data points *k.*

(4) $$y_{axis\;x}=\left|\sum_{i=n}^{k}\omega_{x\left(i\right)}\right|\;y_{axis\;y}=\left|\sum_{i=n}^{k}\omega_{y\left(i\right)}\right|\;y_{axis\;z}=\left|\sum_{i=n}^{k}\omega_{z\left(i\right)}\right|$$

The absolute angular velocity components are kept in a vector defining the ${\vec{y}}_{axis}$ of the rotation matrix, whereas the highest value of the vector indicates the main rotation axis. As this approach aims to detect the main axis of rotation, no prior knowledge of the sensor to foot orientation is necessary [24].

(3) Cross-Product and ${\vec{x}}_{axis}$ determination

The ${\vec{x}}_{axis}$ can be determined by calculating the cross-product:

(5) $${\vec{x}}_{axis}=\;{\vec{y}}_{axis}\times \;{\vec{z}}_{axis}$$

resulting in a vector that is perpendicular to the ${\vec{y}}_{axis}$ and the ${\vec{z}}_{axis}$.

(4) Normalizing and creating of the rotation matrix

After calculating all three axes, each axis has to be normalized. Afterwards, it can be stored in a final rotation matrix:

(6) $$RotationMatrix=\left(\begin{array}{ccc} \hat{x} & \hat{y} & \hat{z} \end{array}\right)$$

After the rotation matrix is determined, the angular velocity and linear acceleration data can be rotated for further processing:

(7) $${\vec{\omega }}_{rotated}=RotationMatrix*{\vec{\omega }}_{current}^{T}$$

(8) $${\vec{a}}_{rotated}=RotationMatrix*{\vec{a}}_{current}^{T}$$

This process is done for each incoming data point. Furthermore, the rotation matrix is recalculated for every new Full Contact, and thus taking variations of individual movements into account.

#### 2.4.2. Gait Event Detection

**Initial Contact:** A gait cycle is initiated by an Initial Contact. When the heel hits the ground, the body needs to be damped and stabilized, either by the muscles or by the robot. This impact results in a peak of the jerk, ${\vec{jerk}}_{InitialContact}$ in the ${\vec{z}}_{axis}$. To detect this event, the following condition is used:

(9) $$jerk_{min}<\left|jerk_{initialContact\;z}\right|<jerk_{max}$$

One has to mention that the jerk during walking and the jerk during robot-assisted gait training varies from step to step. Therefore, a floating threshold is introduced, resulting in a continuous adaption of $jerk_{min}$ and $jerk_{max}$. This floating threshold takes the value from the previous threshold into account and adds a certain percentage of the current jerk in order to achieve higher robustness against outliers. Additionally, a minimum swing-time must be passed before a new Initial Contact can be detected. This is done in order to prevent the false detection of other spikes in the jerk, which may happen during the gait cycle [24].

**Full Contact:** The detection of Full Contact is already realized in the arbitrary sensor alignment algorithm and can be used for gait event detection without any further computational effort.

**Heel Off / Heel Rise:** After the stance phase, Heel Off can be detected. In the terminal stance phase, the ankle extends up to 10 degrees in the dorsal direction [25]. A rotation in the ${\vec{y}}_{axis}$ is induced, causing the heel to rise, which results in an increase of the angular velocity in the main rotation axis $\left(\omega_{HeelOff\;y}\right)$ and an increase of the acceleration ${\vec{a}}_{Heeloff\;}$. Thus, four conditions are used to detect this event:

(10) $$\left|\omega_{HeelOff\;y}\right|<\;\omega_{HeelOff\;tresh\;y}\;\left|a_{HeelOff\;x}\right|<a_{HeelOff\;tresh\;x}\;\left|a_{HeelOff\;y}\right|<a_{HeelOff\;tresh\;y}\;\left|a_{HeelOff\;z}\right|<a_{HeelOff\;tresh\;z}$$

The parameters $a_{HeelOff\;tresh\;x}$, $a_{HeelOff\;tresh\;y}$, $a_{HeelOff\;tresh\;z}$, and $\omega_{HeelOff\;tresh\;y}$ represent threshold values. The acceleration ${\vec{a}}_{HeelOff\;z}$ is gravity corrected under the boundary condition of executing the training on a flat surface.

**Figure 7:**
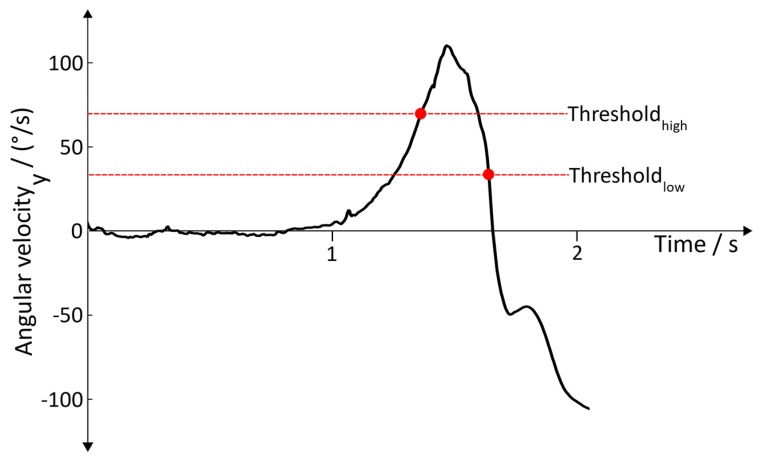
Angular velocity with regard to the ${\vec{y}}_{axis}$ during Toe Off.

**Toe Off:** The pre-swing phase, which follows after the Heel Off, causes a plantar flexion of around 20° in the ankle, followed by reduction of the flexion to around 5° in the initial swing phase and a dorsal extension of the ankle in the middle swing [25]. This motion sequence is characterized by a distinctive change of the angular velocity $\;\omega_{ToeOff\;y}$ of the foot (Figure 7), indicating the lifting off of the toe from the ground.

This motion sequence leads to three conditions:

(11) $$\;\omega_{ToeOff\;y}>\omega_{Threshold_{high}}$$

(12) $$\;\omega_{ToeOff\;y_{\left(i\right)}}<\omega_{y_{\left(i-1\right)}}\;\;\;\;\forall \;i\in \left(n\ldots .k\right)$$

(13) $$\omega_{ToeOff\;y}<\omega_{Threshold_{low}}$$

where *n* denotes the current sample, and *k* denotes a reasonable amount of passed samples. For the Toe Off detection, the conditions (11), (12), and (13) are used and have to be satisfied sequentially

**Figure 8:**
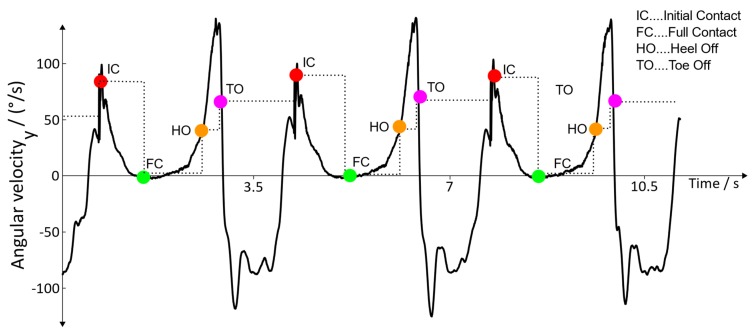
Detected gait cycle.

**Detection of the gait cycle:** For adequate timing of the stimulation, it is crucial that the gait events are detected sequentially. Therefore, they must be passed according to the finite-state diagram depicted in Figure 4. A fully detected gait cycle starts with an Initial Contact and ends with the next Initial Contact of the associated leg. Within these two events, Full Contact, Heel Off, and Toe Off are detected one after another, and the time of detection is stored. If an event is detected outside of the sequence, it is discarded. Thus, the result of the detection algorithm looks as depicted in Figure 8.

#### 2.4.3. Error Handling

Throughout the measuring chain, various errors can occur, most of them resulting in missing data points (e.g.,: losing the Bluetooth connection), false detection of gait events caused by outliers (e.g.,: to high peaks due to an impact that is not associated with the gait pattern), and badly calibrated or defect IMU sensors resulting in erroneous data. These possible errors must be intercepted by the algorithm in order to make the stimulation safe and avoid unwanted stimulation patterns. For error handling, different approaches are implemented. One approach is the previously mentioned finite-state model (Section 2.4). This approach aims to discard wrongly detected gait events. If the previously mentioned sequence is not guaranteed, the step is not considered successful and is not valid. Another approach is focused on the individual gait event detection and aims at detecting correct gait events rather than incorrect ones. This is realized by introducing temporal dependencies of gait events. After the successful detection of an Initial Contact, a minimum roll time must be passed before a Heel Off can be detected. Another temporal dependency is introduced after the Toe-Off event where a minimum swing time must be passed before an Initial Contact can be detected.

#### 2.4.4. Threshold Adaption

Different speeds of the orthosis require adaptive thresholds. Therefore, an angular velocity-dependent adaptation is introduced. To realize that, the maximum angular velocity during Toe Off is detected. Depending on the maximum angular velocity, the temporal dependencies from Section 2.4.3 and the threshold conditions $\omega_{Threshold_{high}}$ from Equation (11) and $\omega_{Threshold_{low}}$ from Equation (13) are multiplied by a certain factor in order to adjust to the current condition.

#### 2.4.5. Robot-Assisted Gait Trainer Adaption

**Figure 9:**
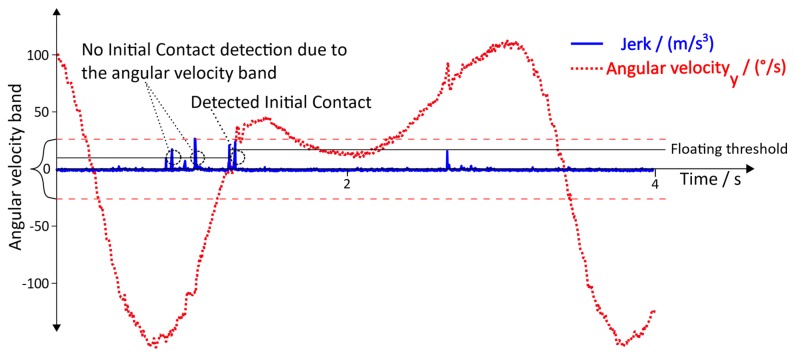
Angular velocity band for Initial Contact (IC) detection.

Both systems (Lokomat and Lyra) claim to provide a physiological gait pattern. Nevertheless, when having a look at the linear acceleration and angular velocity data of the sensors located on a distinct position (Figure 12), the data differ between the systems. This results in robot-assisted gait trainer specific adaptions of the algorithm. In these particular cases, the differences can be identified in various events. The first adaption was done at the Initial Contact detection where the Lyra provides a much lower jerk(m/s^3^) during an Initial Contact, resulting in lower thresholds and different threshold adaption. Another adaption was made for the temporal conditions where the minimum swing time and the minimum roll time were adjusted to the specific needs of the robot. Due to the characteristics of the linear acceleration, the resulting jerk, and the angular velocity data of the Lyra, there was the need to implement an additional error handling condition for the Initial Contact detection. In Figure 9, one can see several spikes in the jerk signal. These spikes may be detected as an Initial Contact, as they happen after the minimum swing time has passed and are high enough to cross the floating threshold. By introducing an angular velocity band, these signals can be discarded, and no Initial Contact is detected.

### 2.5. Functional Electrical Stimulation

For achieving optimal stimulation to restore a physiological gait pattern, the timing of muscle activation is crucial [25]. The amount of involved muscles during walking considering the whole body is tremendous. As stimulation channels are limited and as the system aims to be feasible in clinical routine, the number of stimulated muscles is reduced to the main acting muscles of the lower limb.

**Figure 10:**
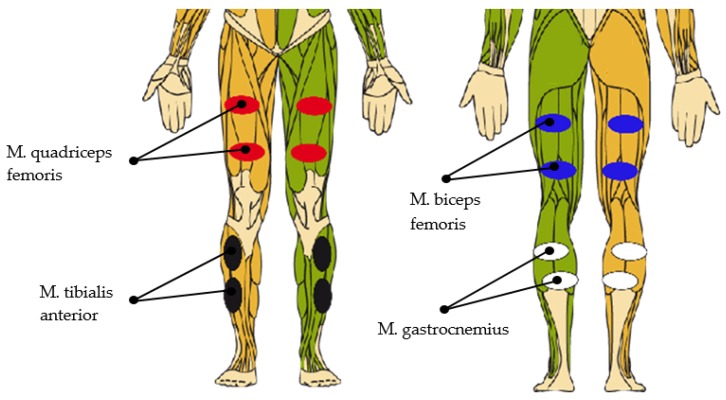
Stimulated muscles and electrode position.

The involved muscles, including their electrode positions in the FES system, can be seen in Figure 10.

Correct timing of the stimulation can be derived from the gait events. Every gait event can be assigned to a certain percentage of the gait cycle (Figure 3), resulting in the chart depicted in Table 1.

For the execution of the stimulation in the future setup, the device RehaStim (chapter 2.2) will be used.

## 3. Results

This section presents the results of the developed algorithm. Section 3.1 deals with arbitrary sensor alignment. Chapter 3.2 is focused on the results of the measurement obtained during robot-assisted gait training.

### 3.1. Arbitrary Sensor Alignment

**Figure 11:**
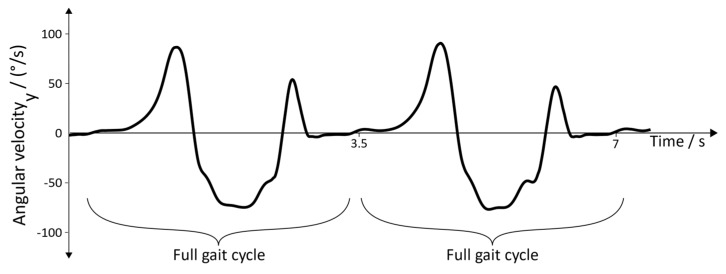
Shape of the angular velocity during two gait cycles.

Arbitrary sensor alignment permits arbitrary mounting of the IMUs, which is crucial for the simplification of the usability, allowing the therapist to focus more on the individual therapy. To make sure that the algorithm works properly, the following properties of the (rotated) sensor data must be satisfied:

- Well-defined shape of the angular velocity of the ${\vec{y}}_{axis}$ (pitch), as depicted in Figure 11
- Acceleration in the ${\vec{z}}_{axis}$ at rest must be around ‒9.81 m/s^2^, which is caused by the gravitational field
- Acceleration in the ${\vec{x}}_{axis}$ and ${\vec{y}}_{axis}$ at rest must be in a low band around zero

As the rotation around the ${\vec{x}}_{axis}$ (roll) and the rotation about the ${\vec{z}}_{axis}$ (yaw) do not provide additional information for the gait event detection, they are not considered in this section.

**Figure 12:**
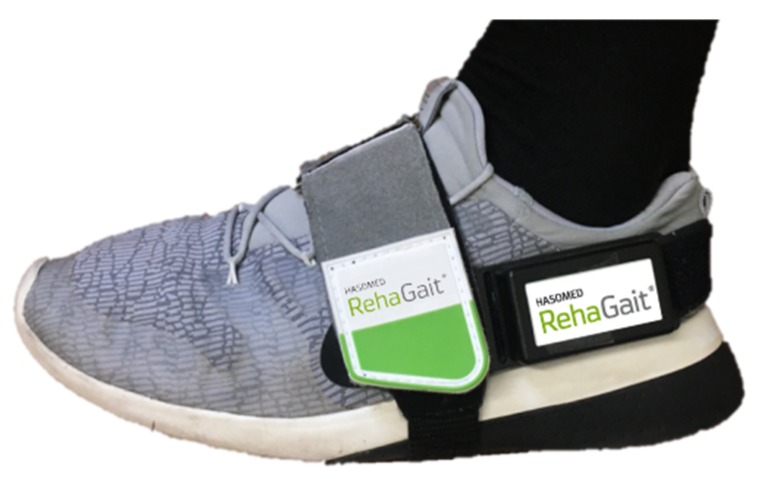
Reference sensor with fixation strap.

**Raw data acquisition**: To avoid synchronization issues, two sensors at the ipsilateral side were used. One sensor was tightly attached to the foot between the calcaneus and the cuboid bone, representing the axis of the foot (reference position); for that purpose, a fixation strap from the company HASOMED GmbH was used (Figure 12).

**Figure 13:**
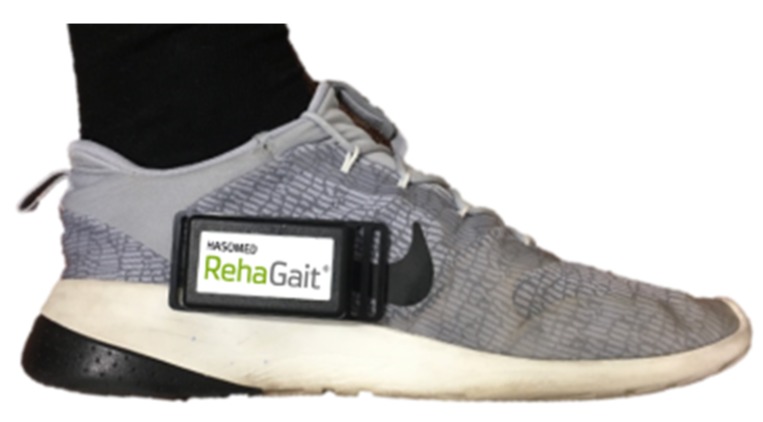
Sensor position: inside of the foot.

**Figure 14:**
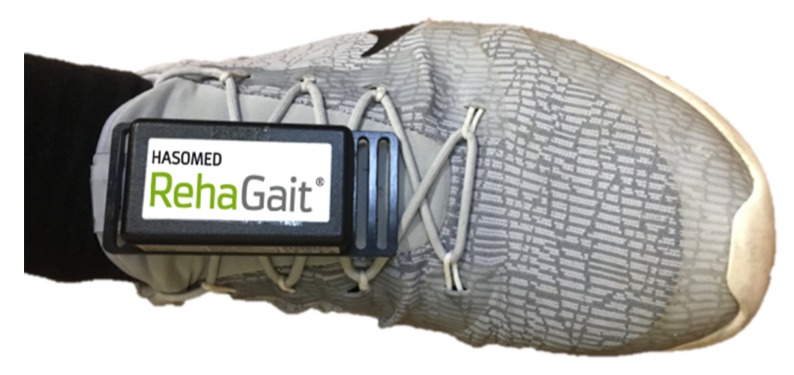
Sensor position: dorsum of the foot.

The second sensor was put on an arbitrary position. Under consideration of the meaningfulness of the placement, the arbitrary sensor position was limited to two positions: the inside of the foot (Figure 13) and the dorsum of the foot (Figure 14).

**Figure 15:**
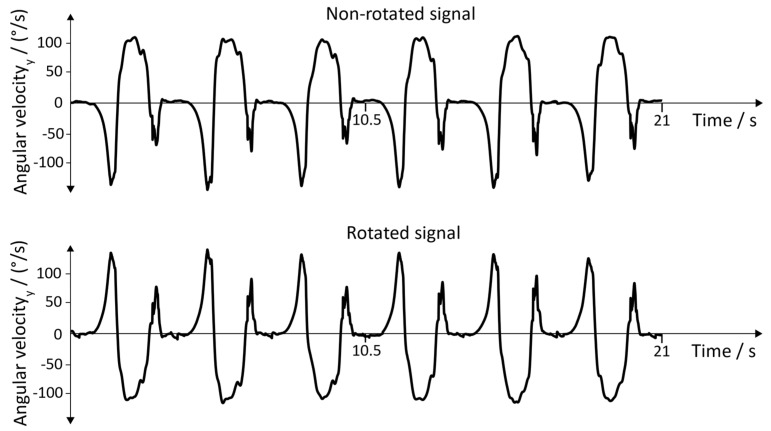
Non-rotated signal and rotated signal of the ${\vec{y}}_{axis}$. Position of the sensor: inside of the foot.

**Figure 16:**
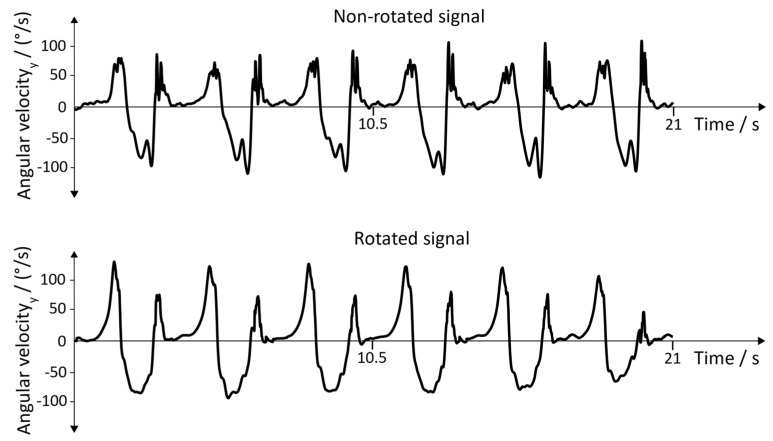
Non-rotated signal and rotated signal of the ${\vec{y}}_{axis}$. Position of the sensor: dorsum of the foot.

**Raw Signals**: The raw signals of the non-rotated and rotated angular velocity data of the sensor with the position “inside of the foot” is shown in Figure 15. Figure 16 represents the angular velocity corresponding to the position “dorsum of the foot”.

**Figure 17:**
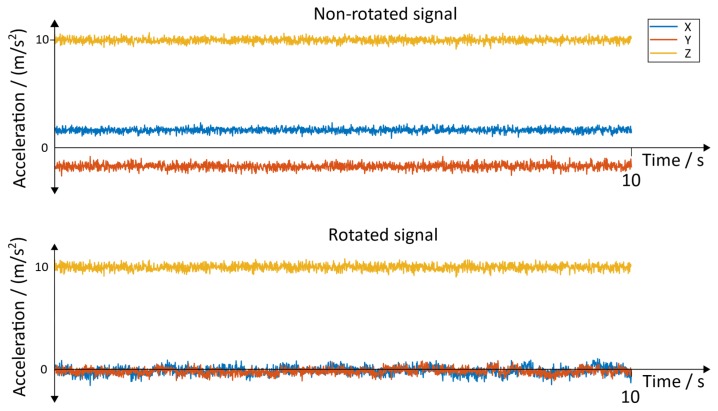
Non-rotated signal and rotated acceleration signals. Position of the sensor: inside of the foot.

**Figure 18:**
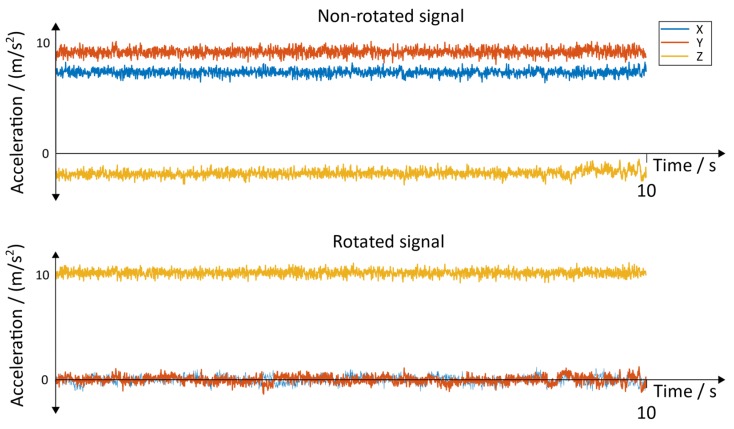
Non-rotated signal and rotated acceleration signals. Position of the sensor: dorsum of the foot.

The non-rotated and rotated linear acceleration data of all three axes of the sensor with the position “inside of the foot” can be seen in Figure 17. The corresponding results for the sensor with the position “dorsum of the foot” can be seen in Figure 18. A resting state of 10 s was chosen to represent the results.

**Normalization (angular velocity:**${\vec{y}}_{axis}$**):** As the distinct shape of the signal should be compared, regardless of the amplitude, the signals need to be normalized, taking the following aspects into consideration:

- Removing of the offset by subtracting the mean value of the signal
- Normalization of the amplitude with the following calculation (written as MATLAB code):
  (14) $$signal_{normalized}=\;\frac{signal_{raw}}{sqrt(max\left(xcorr\left(signal_{raw}\right)\right)}$$

This normalization leads to the fact that the result of the autocorrelation of the signals equals 1, indicating a similarity of 100% and providing a base for comparison.

**Figure 19:**
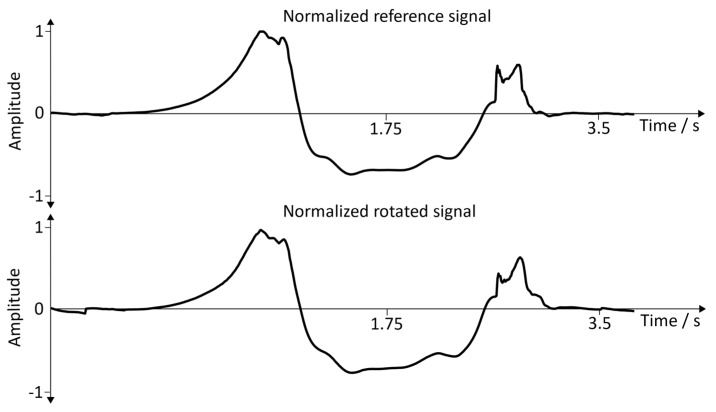
Normalized reference signal and normalized rotated signal. Position of the sensor: inside of the foot.

**Figure 20:**
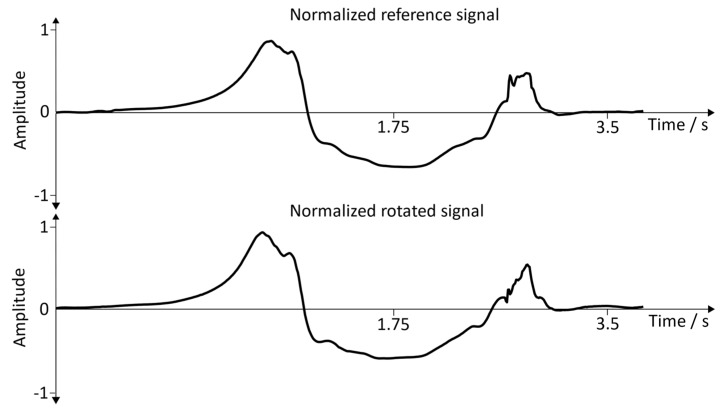
Normalized reference signal and normalized rotated signal. Position of the sensor: dorsum of the foot.

**Normalized signals (angular velocity:**${\vec{y}}_{axis}$**):** For better comparability, a single gait cycle of the reference position and the rotated signal was used. Figure 19 represents the comparisons between the reference signal and the rotated signal with the sensor position: inside of the foot. Figure 20 shows the comparisons between the reference signal and the rotated signal with the sensor position: the dorsum of the foot.

**Results (angular velocity:**${\vec{y}}_{axis}$**):** Based on the normalized angular velocity signals, a cross-correlation was calculated for 20 gait cycles. This allowed to compare the shape of the signal for each step individually, and thus verifies whether the rotation was successful. This comparison was made in order to verify the functionality of the arbitrary sensor alignment algorithm before the recording sessions (Section 3.2 and Section 3.3) were executed. The mean and the standard deviation for all 20 cross-correlations were calculated, and the corresponding results can be seen in Table 2.

**Results (acceleration):** For the acceleration, all the resting phases within the above-mentioned 20 gait cycles were considered. The resting phase was chosen as it is the only phase during the gait cycle where an assumption of the acceleration of all three axes can be made. During an ideal resting phase, the rotated ${\vec{z}}_{axis}$ of the acceleration data is influenced by the gravitational field, and thus has a value of around 9.81 m/s^2^ (gravitational acceleration). The other two axes should ideally be 0, as under perfect conditions, the IMU does not move during a resting position. As the measurements were executed under normal conditions and can thus not fulfill ideal conditions, small deviations (e.g.,: noise of the sensor) are considered as normal. To check whether the rotation was successful, the mean value and the corresponding standard deviation of the resting phases were calculated (Table 3).

### 3.2. Lokomat Measurements

**Figure 21:**
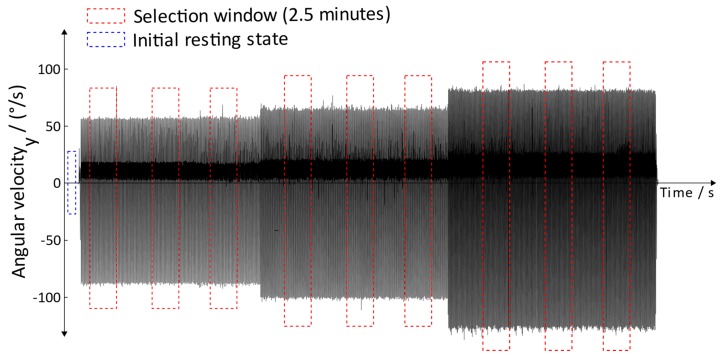
Selection method for analyzing data.

The Lokomat measurements were executed at the MEDIAN neurological rehabilitation center in Magdeburg. The orthosis of the Lokomat was adjusted and checked by a specially trained physiotherapist; thus, an optimal training setup was provided. To avoid unwanted support of the muscles of the subject, the executive force of the Lokomat was set to a maximum. This boundary condition guaranteed that the recorded movement data corresponded to the movements initiated by the Lokomat rather than the movement of the subject. Three recording sessions were performed by a 39-year-old healthy male adult. Each recording session had a duration of 60 min, including setup time. The executed velocities (1.2, 1.5, and 1.7 m/s) represent typical velocities used for rehabilitation. During the Lokomat training, the movement was recorded using IMUs (Section 2.3). The IMUs were attached to the foot of the subject in order to detect the gait phases. The data was analyzed using the above-mentioned gait detection algorithm. For the analysis, a window of 75,000 data points (equal to 2.5 min) was used; within this window, the detection rate was calculated. Three windows were chosen for each velocity, resulting in a total amount of nine windows and 675,000 analyzed data points (Figure 21).

This approach was used in order to generate a repeatable and comparable analyzing process. It was executed once for each recording session. The start of the windows was chosen to be a resting phase of a gait cycle; the according end of the window was determined by the size of the chosen window. The detection rate represents the correctly detected steps and is calculated as follows:

(15) $$Detection\;rate\;=\;\frac{100}{\;steps_{reference}}*steps_{algorithm}$$

**Figure 22:**
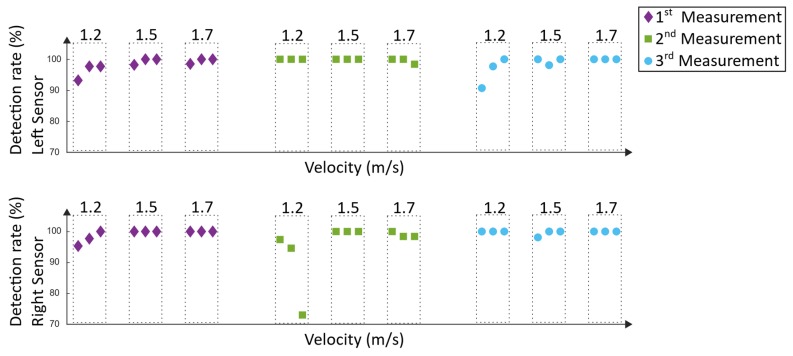
Detection rate of the Lokomat measurements.

The detection rate in Equation (15) was calculated for each window by determining the amount of correctly detected steps of the algorithm ($steps_{algorithm}$ = $steps_{detected}$ − $steps_{incorrect\;detected}$), whereas the manually counted steps served as a reference ($steps_{reference}$). In Figure 22, the results of the detection rate can be seen.

**Figure 23:**
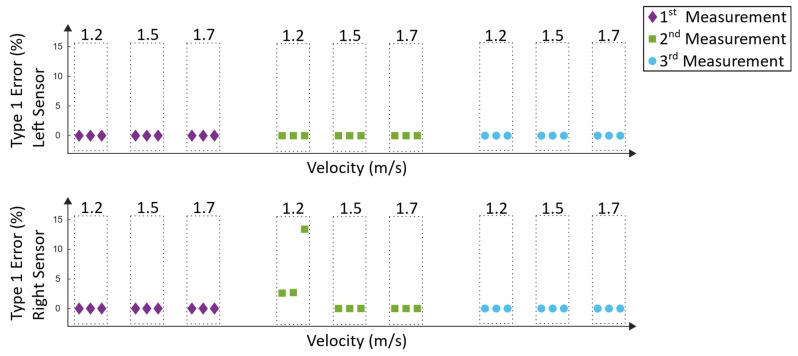
Type-1 errors of the Lokomat measurements.

Type-1 errors (Figure 23) represent incorrectly detected steps. These errors can trigger incorrect timing in the electrical stimulation and thus cause a potential hazard for the subject, whereas not-detected steps (type 2 errors) are not considered hazardous. The type-1 error was calculated as follows:

(16) $$Type\;1\;error=\;\frac{100}{\;steps_{reference}}*steps_{incorrect\;detected}$$

The type-1 error in Equation (16) was calculated for each window by determining the amount of incorrect detected steps of the algorithm ($steps_{incorrect\;detected}$), whereas the manually counted steps served as a reference ($steps_{reference}$). Incorrected steps are steps that do not represent the typical pattern in the movement data. In Figure 23, the according results can be seen.

### 3.3. Lyra Measurements

**Figure 24:**
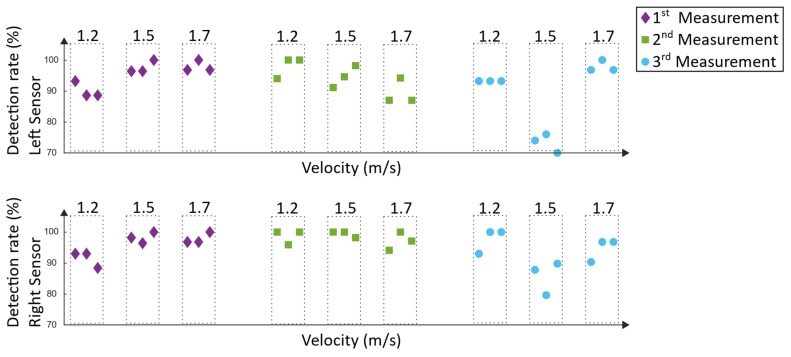
Detection rate of the Lyra measurements.

The Lyra measurements were executed at the NRZ rehabilitation center in Magdeburg. The foot support of the end-effector based system and the body weight support system were adjusted and checked by two specially trained physiotherapists; thus, an optimal training setup was provided. Three measurements were carried out, and executed by the same 39-year-old male adult as during the Lokomat measurements. Each training had a duration of 60 min, including setup time. The same velocities as during the Lokomat training were tested, and the same approach as stated in Section 3.2 was used to analyze the data. In Figure 24, the detection rates can be seen.

**Figure 25:**
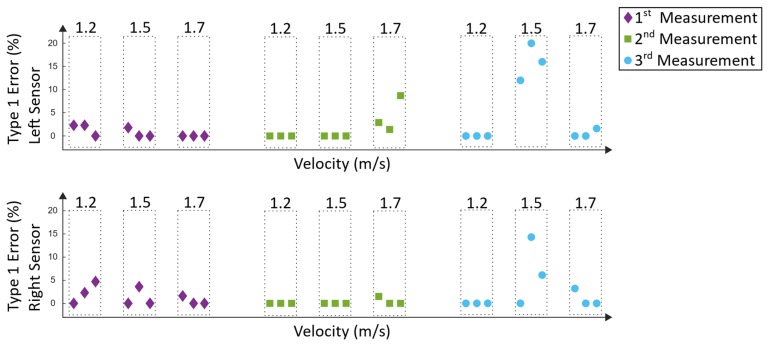
Type-1 errors of the Lyra measurements.

Similar to the analysis of the Lokomat measurements, the type-1 errors of the Lyra system (Figure 25) represent the incorrected detected steps, which could trigger hazardous electrical stimulation.

## 4. Discussion

The discussion section aims to evaluate the results of Section 3. In order to get a better overview of the results reported in Section 3, the arbitrary sensor alignment, the Lokomat measurements, and the measurements done at the Lyra will be evaluated separately.

### 4.1. Arbitrary Sensor Alignment

The arbitrary sensor alignment is an essential part of the gait detection algorithm. It is one of the main components to guarantee an easy setup, as the clinician who will be using the technique in future does not have to stick to a pre-defined position of the sensor. Additionally, the arbitrary sensor alignment is one of the first computations that the algorithm executes. This leads to the fact that all the other calculations are dependent on a correctly executed rotation of the sensor data. As the angular velocity in the ${\vec{y}}_{axis}$ and the acceleration in all three axes are of importance, these values were analyzed in Section 3.1. The result from the cross-correlation comparing the reference signal with the rotated signal of the sensor at the position “inside of the foot” is 0.988 ± 0.012. Comparing the reference signal to the signal of the sensor at the “dorsum of the foot” results in a value of 0.990 ± 0.006 (Table 2). As both of these signals have a value close to one, which would mean that the signals have the exact same shape, the results are satisfying. As mentioned in Section 3.1, when assuming ideal conditions, the acceleration values of the ${\vec{x}}_{axis}$ and the ${\vec{y}}_{axis}$ would have a value of 0. These ideal conditions are not achievable in practice, as slight movements of the sensor or the foot and the noise of the sensor influence the data. Taking these aspects into consideration, the results of the ${\vec{x}}_{axis}$ and the ${\vec{y}}_{axis}$ are satisfying, as they are all close to 0. The same counts for the results of the ${\vec{z}}_{axis}$, which both provide a result close to 9.81 m/s^2^ (Table 2). Based on these findings, the arbitrary sensor alignment algorithm works as intended, and provides good result for the further computation of the signal.

### 4.2. Lokomat Measurements

In order to evaluate the results of the Lokomat measurements, the detection rate (Figure 22) and the type-1 errors (Figure 23) must be considered. As mentioned in Section 3.2, type-1 errors are considered hazardous as they might trigger a stimulation that does not correspond with the current phase of the gait cycle. Except for one measurement (second measurement at velocity 1.2 m/s), all measurements have a type-1 error of 0% and a detection rate between 90.7% and 100%. During the second measurement at 1.2 m/s, the lowest detection rate was 73%. The type-1 error was up to 13.4% at the right sensor, which would not be tolerable during a Lokomat training. As this was the only measurement and the only velocity where a type-1 error happened, the explanation for that could be that adjustments in the setup during the training have been done. In particular, the body weight support system was adjusted as the subject reported discomfort. This could have resulted in movement in the legs and had a resulting influence on the data. Another explanation could be that the discomfort per se lead to a voluntary movement of the subject, which could have resulted in an influence on the robot-induced gait pattern. Overall, a mean detection rate of 98.1% ± 5.2% could be achieved. The mean type-1 error was 0.3% ± 1.2%. Approaches using IMUs for gait-event detection of human walking achieved similar detection rates [26,27]. However, these approaches aim to detect the human gait and not robot-specific gait events.

### 4.3. Lyra Measurements

The detection rate and the type-1 errors of the Lyra measurements can be seen in Figure 24 and Figure 25. The first measurement revealed detection rates between 88.6% and 100%. Type-1 errors range from 0% up to 4.7%. As mentioned before, type-1 errors are considered hazardous; nevertheless, this small amount could be tolerable and could be diminished by additional error handling. Similar results were found in the second measurement, where the detection rates were between 87% and 100%. The highest type-1 error was 8.7%. The results of the third measurement were similar, except for one outlier during 1.5 m/s. During this recording session, the worst results were acquired. The lowest detection rate was 70% and the highest type-1 error was 20%. These values are not tolerable, as they would not support the rehabilitation process of the subject. The reason for this result could be that the subject tried to re-adjust the body weight support system. This resulted in left and right oscillations of the subject’s trunk and in a resulting movement of the lower extremities. Despite the rigid fixation of the Lyra, large movements of the subject can influence the movement of the lower extremities, and thus may result in unwanted disturbances in the induced gait pattern. Overall, a mean detection rate of 94.1% ± 6.8% could be achieved. The mean type-1 error was 1.9% ± 4.3%. As mentioned before, comparable approaches using IMUs to detect gait events of human walking revealed similar detection rates of about 99% [26,27].

## 5. Conclusions

Based on the results discussed in the previous sections, a system using inertial measurement units for gait event detection of robot-assisted gait training is feasible; thus, a system as conceptually shown in Figure 1 and Figure 2, which aims to support Lokomat or Lyra training with functional electrical stimulation, is feasible as well. The easy setup of two IMUs allows fast setup times. Additionally, the arbitrary sensor alignment algorithm guarantees versatile sensor positions and partially prevents wrong data acquisition due to misplacement of the sensor. The results of the gait event detection algorithm largely provide promising results for further research and for the development of the proposed system. High type-I errors could be the results of subject-induced movement. Further measurements to investigate this issue should be executed. To avoid subject-induced type-I errors, further error-handling methods should be implemented. One of the limitations was that the measured Lokomat and Lyra trainings were executed by a healthy subject. Despite the chosen boundary condition of maximum execution force of the Lokomat, it may be the case that the healthy subject influenced the gait pattern. In contrast to the Lokomat, the executive force was not adjustable at the Lyra; nevertheless, similar effects could have influenced the movement pattern. To check whether this is the case, measurements with patients who are suitable for gait rehabilitation with a Lokomat or a Lyra should be executed for further evaluation. Type-2 errors are not considered hazardous, as steps that are not detected would not trigger a stimulation; nevertheless, they should be investigated in future research in order to enhance the overall detection rate.

Robot-assisted gait trainers detect the patient output [28]. Patient output can be used to detect the spasticity and voluntary movement of the patient. If the robot detects spasticity, the robotic movement is stopped in order to prevent injuries. The stopped movement of the gait trainer would result in linear acceleration and angular velocity data, which would not be identified as step. The error-handling methods described in Section 2.4.3 would discard these events, and no electrical stimulation would be triggered. Additionally, joint contractures or limitations in the range of motion due to spasticity are considered as a risk factor for robot-assisted gait training and must be carefully evaluated by health care professionals before the rehabilitation. Nevertheless, the influence of spasticity has not been tested in detail as no patient with neurological deficits was measured so far. Further measurements including patients suffering from neurological deficits are needed to address this topic.

The described concept offers the possibility of stimulating the muscles according to the most important gait events (Figure 4). As a result, changes of the gait pattern during the gait training can be detected, and stimulation can be finely adjusted. Other approaches initiate the stimulation based on Initial Contact [11], the start and end phase of the stance period [13] or were triggered using a finger switch [12] which results in limited flexibility. In contrast to other approaches [11,13] this concept uses inertial measurement units for data acquisition, which makes it independent to the information provided by the robot-assisted gait trainer. Thus, the concept can be extended for use with various other robot-assisted gait trainers. Additionally, the electrical stimulator (2.2) uses surface electrodes compared to other approaches where intramuscular electrodes were used [11,12].

The presented algorithm works as intended; nevertheless, it is important to mention that no validation with gold standards has been done. Despite some limitations, the presented concept offers a novel and feasible approach that could enhance the rehabilitation process of robot-assisted gait training with functional electrical stimulation.

## Figures and Tables

**Table 1:** Timing of stimulation, adapted from [25]

Muscle	Start (% of Gait Cycle)	Stop (% of Gait Cycle)
M. quadriceps femoris (left)	90	16
M. quadriceps femoris (right)	40	66
M. biceps femoris (left)	80	12
M. biceps femoris (right)	30	62
M. tibialis anterior (left)	56	12
M. tibialis anterior (right)	6	62
M. gastrocnemius (left)	10	50
M. gastrocnemius (right)	60	100

**Table 2:** Results: angular velocity data in the y-direction (cross-correlation).

Number of Gait Cycles	Sensor Position	Mean	Standard Deviation
20	Inside the foot	0.988	0.012
20	Dorsum of the foot	0.990	0.006

**Table 3:** Results: acceleration data.

Number of Resting Phases	Sensor Position	Axis	Mean (m/s^2^)	Standard Deviation (m/s^2^)
20	Inside the foot	x_axis_	−0.142	0.197
20	Inside the foot	y_axis_	0.108	0.246
20	Inside the foot	z_axis_	9.944	0.110
20	Dorsum of the foot	x_axis_	−0.514	0.273
20	Dorsum of the foot	y_axis_	0.200	0.323
20	Dorsum of the foot	z_axis_	10.028	0.126
